# Applications of Chitosan-Alginate-Based Nanoparticles—An Up-to-Date Review

**DOI:** 10.3390/nano12020186

**Published:** 2022-01-06

**Authors:** Adelina-Gabriela Niculescu, Alexandru Mihai Grumezescu

**Affiliations:** 1 Department of Science and Engineering of Oxide Materials and Nanomaterials, Faculty of Applied Chemistry and Materials Science, Politehnica University of Bucharest, 011061 Bucharest, Romania; adelina.niculescu@upb.ro; 2 Research Institute of the University of Bucharest—ICUB, University of Bucharest, 050657 Bucharest, Romania; 3 Academy of Romanian Scientists, Ilfov No. 3, 050044 Bucharest, Romania

**Keywords:** chitosan, alginate, polysaccharide nanoparticles, chitosan-alginate nanoparticles, biomedical applications, drug delivery systems

## Abstract

Chitosan and alginate are two of the most studied natural polymers that have attracted interest for multiple uses in their nano form. The biomedical field is one of the domains benefiting the most from the development of nanotechnology, as increasing research interest has been oriented to developing chitosan-alginate biocompatible delivery vehicles, antimicrobial agents, and vaccine adjuvants. Moreover, these nanomaterials of natural origin have also become appealing for environmental protection (e.g., water treatment, environmental-friendly fertilizers, herbicides, and pesticides) and the food industry. In this respect, the present paper aims to discuss some of the newest applications of chitosan-alginate-based nanomaterials and serve as an inception point for further research in the field.

## 1. Introduction

The development of nanotechnology has led to disruptive applications in many fields, including medicine, biology, electronics, energy, agriculture, and the food industry [1,2,3,4,5,6]. Various materials started being reduced to nanoscale dimensions and tailored to meet the exact requirements of specific applications. Particularly, polymers have been noted for their variety, versatility, and tunability, attracting extensive research efforts in their translation to nanomaterial formulations of use in diversified domains [7,8,9].

In biomedicine, of special interest are natural polymeric nanoparticles (NPs) that can be used as vehicles for the controlled and sustained delivery of a broad range of therapeutics without raising toxicity concerns. Thus, numerous polysaccharide and protein-based nanosystems have recently emerged as potential drug delivery platforms. Out of the plethora of possibilities, marine-sourced carbohydrates are among the most researched materials for biomedical purposes, benefiting from wide availability, abundance, simplicity of fabrication, biocompatibility, biodegradability, and ease of functionalization [10,11,12,13]. Chitosan and alginate are two of the most studied polymers of this sort, being the base materials for a multitude of drug carriers, wound dressings, and tissue engineering scaffolds [14,15,16,17,18,19]. Despite their individual uses, chitosan and alginate have been recently investigated together as a strategy to overcome the limitations of each material, produce synergistic outcomes, and expand their range of applications.

This paper aims to briefly present the properties of interest of chitosan, alginate, and chitosan-alginate nanostructures as argumentation of their recent extensive research, further focusing on their novel uses. In this respect, an overview of their biomedical applications has thoroughly gathered the recent advances in chitosan-alginate-based drug delivery systems, antimicrobial nanoparticles, and vaccine adjuvants. Moreover, other applications are considered, including reducing water pollution, developing better solutions for agriculture, and designing innovative food additives.

## 2. Individual and Synergic Properties

Chitosan is a Food and Drug Administration (FDA)-approved biopolymer with excellent biological properties, including antitumor, antioxidant, antimicrobial, and wound healing activities, that render it suitable for biomedical applications. Its biocompatibility, biodegradability, nontoxicity, mucoadhesivity, and hemocompatibility are ideal characteristics for designing nanocarriers with controlled delivery and sustained release of various therapeutics [10,20,21,22,23,24,25,26,27]. Chitosan has shown great promise in nanodelivery for the treatment of cancer [28,29,30], diabetes [31,32,33], eye diseases [34,35,36], infectious diseases [37,38,39,40,41], and more, being also recently employed in the delivery of vaccines [42,43,44,45].

**Figure 1:**
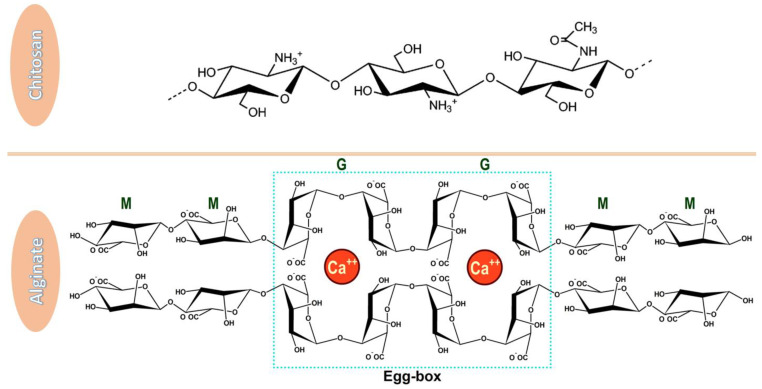
Chemical structures of chitosan and alginate. Chitosan has a linear structure comprised of β–(1-4) linked 2-acetamide-2-deoxy-d-glucose and 2-amino-2-deoxy-d-glucose [49], while alginate is a copolymer of β-D-mannuronic acid (M) and α-L-guluronic acid (G) which, in the presence of calcium ions form an egg-box structure [50]. Adapted from open-access sources.

Moreover, its structure is abundant in modifiable functional groups (i.e., hydroxyl, amino, and carboxyl groups) (Figure 1), allowing chemical modifications towards enhancing water solubility, increasing targeting, and improving stability [10,46,47]. Specifically, the insolubility of chitosan at physiological pH can be solved by additional deacetylation or chemical modification of amino groups leading to soluble derivatives and expanding the applications of this already versatile material [48]. In addition, the stability of this polysaccharide can be enhanced by controlling the environmental factors, launching a proper stabilizing compound, creating blends with other polymers (e.g., alginate, polylysine, poly(γ-glutamic acid), and short-chain amylose) or altering its structure with chemical/ionic agents [25,46].

One of the most convenient natural polymers for forming blends with chitosan is alginate (Figure 1), especially because its anionic nature complements the cationic backbone of chitosan towards forming a more stable nanomaterial. Alginate is also known for desirable biological and physicochemical characteristics, being a profitable FDA-approved material for the development of drug delivery systems [51]. It is a highly available, low-cost, renewable, non-toxic, biodegradable, and biocompatible material that can be further improved through chemical or physical modifications. Alginate also exhibits mucoadhesive properties and, in contrast to chitosan, it is also water-soluble. In addition, studies have shown that alginate-based substances are pH-sensitive, being a useful tool for controlling the release of encapsulated biomolecules [26,47,52,53,54]. Alginate is a promising candidate material for the oral, ocular, nasal, parenteral, and mucosal drug delivery of various moieties, including nucleic acids, drugs, and vaccine formulations [55,56,57,58,59].

Furthermore, alginate generally shrinks at low pH and dissolves at high pH values. Contrarily, chitosan dissolves at low pH, while at high pH values becomes insoluble. Thus, creating polyelectrolyte complexes between these two materials has become of important research interest for overcoming the limitations of each material [60,61]. Adding chitosan in alginate-based nanoparticulate formulations can prolong the contact time of active ingredients with the epithelium, and the absorption can be enhanced by opening the intracellular tight junctions [25,62]. Moreover, chitosan and alginate work synergistically to protect the loaded biomolecules from oxidation, enzymatic degradation, and hydrolysis, ensuring safe and effective delivery to the desired tissues or organs [63].

## 3. Preparation Methods

**Figure 2:**
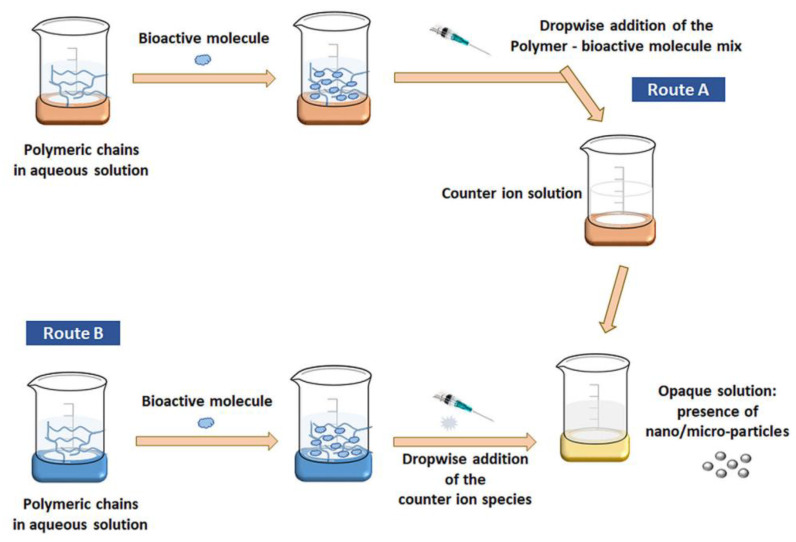
Schematic representation of nanoparticles production via IG method. Reprinted with permission from [64], ^©^ 2020, Society of Chemical Industry, Published by John Wiley and Sons.

Several techniques have been reported in the literature concerning the synthesis methods of chitosan-alginate nanomaterials. One of the most frequently utilized production methods is ionotropic gelation (IG) (Figure 2). IG is based on the electrostatic interactions between two ionic species that, under certain conditions, can produce nanoparticles [64,65]. Drug-encapsulated beads can also be formed through IG by dropping a drug-loaded polymeric solution into the aqueous solution of polyvalent cations. Similarly, biomolecules can also be entrapped in the polysaccharide beads under mild conditions, preserving their three-dimensional structure [66]. IG is frequently used in nanoparticle production due to many advantages, including high encapsulation efficiency, cost-effectiveness, and bio-safe protocols. Nonetheless, this technique may lead to particle size heterogeneity, requiring controlled stirring and a constant speed of solution dropping [64].

The electrostatic interactions between chitosan and alginate lead to the production of chitosan-alginate nanoparticles (CANPs) with a chitosan core and a chitosan-alginate surface. However, studies have reported that the stability of the interaction can be increased by modifying the IG procedure with the addition of a salt. In this manner, there could be employed a pre-gelation phase (between one of the polysaccharides and the salt) and a polyelectrolyte complexation phase (when the second polysaccharide is added to the reaction) [64,66].

Polyelectrolyte complexation assumes strong electrostatic interactions between oppositely charged particles, being a convenient alternative to the use of chemical crosslinking agents. More specifically, these physical interactions occur between the negatively charged carboxylic acid groups from alginate and the positively charged amino groups from chitosan [65,67]. In this way, there can be obtained alginate beads with a polyelectrolyte complex on their surface [66].

Alternatively, CANPs can be obtained through water-in-oil emulsification. This is considered a more complex production method, but it is recognized for enhancing control over particle size and particle size distribution. These features are important when uniform particles must be obtained for ensuring repeatable, controlled release behavior [68].

Other preparation techniques include, but are not limited to, sonication [69,70], electrospraying [71], electrostatic gelation [72,73,74], extrusion of polymer dispersions [75], self-assembly of polysaccharides [65], and microfluidic methods [76,77].

## 4. Biomedical Applications

### 4.1. Drug Delivery Systems

**Figure 3:**
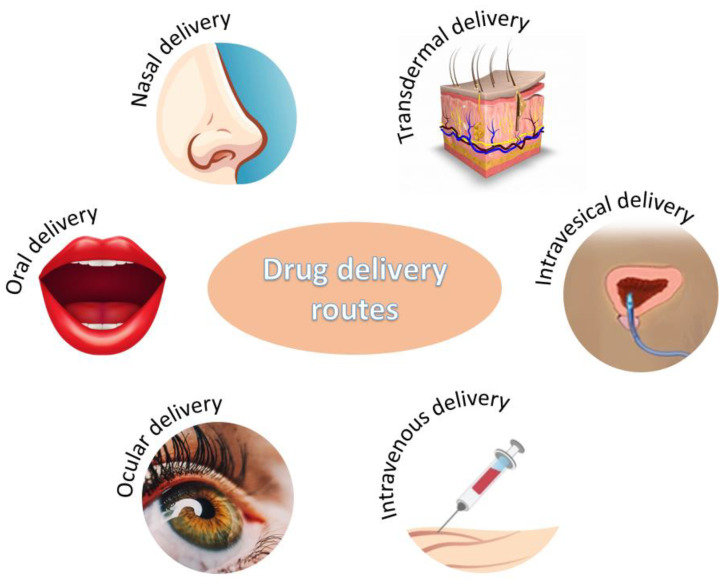
Administration routes for chitosan-alginate-based drug delivery systems.

The unique properties of CANPs make them suitable for delivering a wide range of therapeutics through various administration routes (Figure 3), depending on the treated condition and targeted delivery site.

#### 4.1.1. Oral Delivery

Oral drug administration is one of the most common, convenient, and comfortable routes for patients [25]. However, directly administering drugs via the oral route may face several biological or technical obstacles. Specifically, the biological environments through which the active ingredient passes before reaching its target can interact with the drug leading to its denaturation or preventing effective absorption in the desired tissues. On the other hand, technical issues revolve around finding proper methods for avoiding biological barriers [78]. In this context, chitosan-alginate nanoparticles represent a viable solution for protecting orally administered drugs, reducing systemic toxicity, and enhancing cellular uptake at the target site.

CANPs have been reported useful for the encapsulation of chemotherapeutics. Sorasitthiyanukarn et al. have successfully incorporated curcumin diethyl diglutarate [79] and curcumin diglutaric acid [80] in chitosan-alginate-based nanocarriers. The scientists obtained promising results as the encapsulation in polysaccharide particles improved in vitro digestibility and bioaccessibility under simulated gastrointestinal conditions, ensured controlled drug release, increased cellular uptake in cancer cells, and enhanced anticancer activity of the drugs.

**Figure 4:**
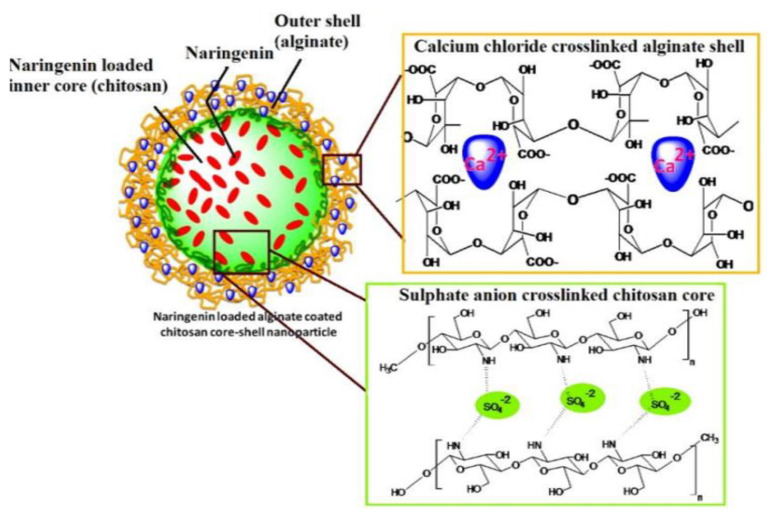
Schematic representation of the delivery system developed by Maity et al. [69]. Reprinted with permission from [69]. ^©^ 2017, Elsevier Ltd.

Another important application of CANPs is the treatment of diabetes mellitus. Polysaccharides have found uses in oral insulin administration as an alternative to subcutaneous injection, aiming to decrease the associated discomfort and increase patient compliance [81,82,83]. Chitosan-alginate-based delivery systems can also be employed for diabetes treatment when encapsulated with other drugs. For instance, Mukhopadhyay et al. [84] have designed quercetin-succinylated chitosan-alginate core-shell-corona NPs that showed pronounced hypoglycemic effect and efficient glucose homeostasis maintenance. Alternatively, Maity et al. [69] have prepared naringenin-loaded alginate-coated chitosan core-shell NPs (Figure 4) that could effectively deliver the flavonoid and reduce the glycemia of tested animals without raising toxicity concerns.

Other emerging applications of CANPs-based oral delivery systems include improving solubility and therapeutic effects of antilipidemic drugs [85], protecting against oxidative stress [72,73], potentiating the treatment of obesity, cardiovascular diseases [86], and multiple sclerosis [87], and enhancing the bioavailability of active compounds in functional foods, nutraceuticals, and dietary supplements [88].

Several relevant examples of CANPs for oral drug delivery are described in Table 1 from the points of view of their synthesis method, physicochemical properties, and potential biomedical application.

#### 4.1.2. Ocular Delivery

The eye is a highly sensitive organ, being exposed to both environmental harm and internal damaging factors, such as age, genetics, and diabetes. Nonetheless, the eye is protected by several anatomic and physiological barriers (e.g., blinking, different membraneous layers, blood-retinal barrier, choroidal and conjunctival blood flow, lymphatic clearance, lacrimation) that limit the supply of drugs to affected tissues [71,89,90,91]. Thus, developing nanocarriers for ocular delivery became necessary for improving drugs bioavailability, controlling drug release, decreasing the frequency of administrations, and increasing therapeutic efficiency [71,92,93].

In this context, chitosan and alginate are ideal base materials for ocular drug delivery vehicles due to their favorable properties, including superior biocompatibility, bioadhesion, permeability-enhancing properties, ability to prolong drug’s residency time, and capacity for sustained drug release [94,95]. Consequently, researchers started investigating various chitosan and alginate-based nanosystems, recently focusing on different combinatorial approaches for treating eye diseases (Table 2).

#### 4.1.3. Other Drug Delivery Systems

Despite not benefiting yet from the same extensive research efforts as oral and ocular delivery systems, chitosan-alginate nanoparticles have been reported promising for other administration routes as well.

The mucoadhesive properties of these polymers are considered advantageous for nasal drug delivery. For instance, Lee et al. [102] have chosen CANPs for the encapsulation of *Apis mellifera* bee venom due to these polysaccharides’ slow-releasing properties and bioadhesion. The authors reported that their newly developed system could be applied as a preventive and therapeutic agent against porcine reproductive and respiratory syndrome virus (PRRSV). Its nasal delivery leads to a significant decrease of viral burden in the sera and tissues of the tested pigs and non-specific immune-stimulating actions.

Respiratory infections can also be tackled via intratracheal administration of drugs encapsulated in CANPs. Scolari et al. [103] have approached this strategy to co-deliver rifampicin and ascorbic acid towards attaining enhanced biocide activity against *S. aureus*. Furthermore, the researchers reported synergic effects between the carrier and the loaded antibiotics, concluding that this delivery platform is suitable for antibiotic lung administration in antimicrobial-resistant infections.

The ability of CANPs to enhance skin penetration of drugs was exploited by Abnoos et al. [104], who have encapsulated pirfenidone (PFD) for transdermal administration. In this way, the scientists avoided the pharmacotherapeutic limitations of orally administered PFD, obtaining 94% efficiency in treating pulmonary fibrosis.

CANPs have also been evaluated for the encapsulation of drugs administered by intravenous injections. Yoncheva et al. [105] have used these carriers for loading doxorubicin as a novel delivery system for melanoma treatment, while Hashemian et al. [70] incorporated curcumin and tested the nanosystem in epilepsy treatment.

**Figure 5:**
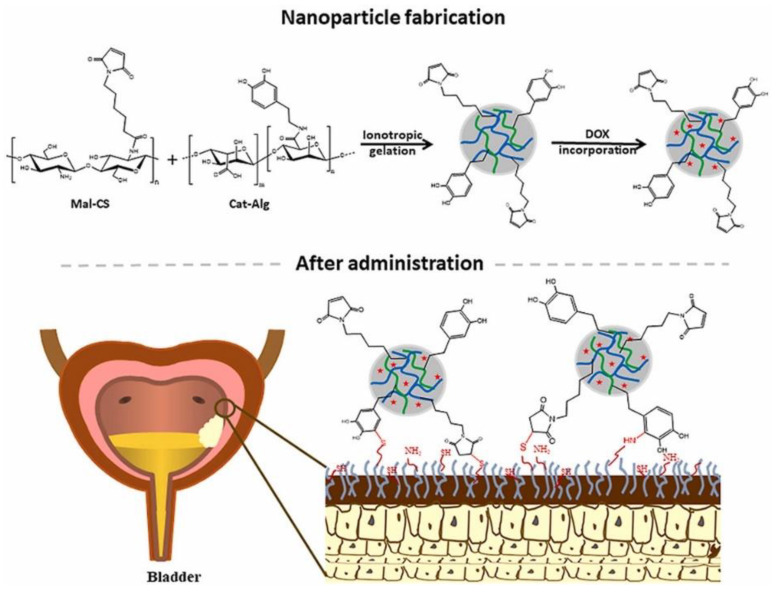
Schematic representation of the synthesis mechanism and final structure of the delivery system developed by Sahatsapan et al. [106]. Reprinted with permission from [106], ^©^ 2021, Elsevier B.V.

Another interesting delivery approach is employing chitosan and alginate-based carriers for intravesical drug administration. As an example, Sahatsapan et al. [106] have used maleimide-bearing chitosan (Mal-CS) and catechol-bearing alginate (Cat-Alg) to create NPs with dual mucoadhesive moieties for doxorubicin encapsulation (Figure 5). The authors reported potent inhibitory effects against mouse bladder carcinoma cell line MB49, with sustained doxorubicin release and enhanced cell uptake, concluding that the designed NPs represent promising delivery vehicles for bladder cancer treatment.

A different strategy reported in the literature is the use of chitosan-alginate-based vehicles for the local delivery of probiotics in treating periodontal disease. For instance, Mirtič et al. [75] have designed probiotic-loaded microcapsules that deliver their cargo within periodontal pockets, promoting their prolonged survival, efficient revival, and successful colonization of the target surface.

For clearance, the above-discussed delivery systems have been summarized in Table 3.

In addition to the above-discussed studies, important results have been registered for various other chitosan-alginate-based delivery systems, which, however, have not yet been established for a particular administration route. Such innovative systems are currently in the in vitro testing stage for application in the treatment of different infectious and chronic diseases (Table 4).

Out of the potential applications of chitosan-alginate-based nanomaterials, anticancer formulations have benefited from increased research interest. These polysaccharides have been used due to their biocompatibility, high loading capacity, and appealing biological properties. Particularly, they are considered advantageous due to their ability to ensure controlled and targeted drug release, diminishing the systemic adverse effects associated with carried anticancer drugs. Moreover, the intrinsic antitumor, antioxidant, and pH-responsive character of these polymer nanocomposites recommend them for enhancing treatment outcomes by improving cancer cell uptake and working synergically with the loaded therapeutics [80,109,111].

**Figure 6:**
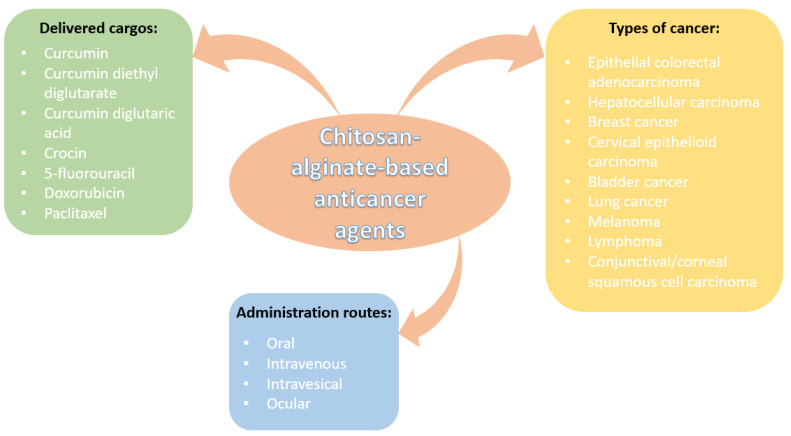
Overview of chitosan-alginate-based anticancer nanoformulations.

As it can be noticed from the numerous mentioned studies in the field, CANPs are carriers of choice for encapsulating chemotherapeutic agents to be delivered through various administration routes towards treating different types of cancers. Figure 6 highlights the importance of chitosan-alginate-based nanoconstructs and visually synthesizes the above-presented information.

Moreover, recent scientific interest has been drawn to creating smart delivery systems by integrating biodegradable polymers with drugs and metal-based nanoparticles with magnetic properties. In this respect, Song et al. [114] have proposed the encapsulation of curcumin into magnetic alginate-chitosan NPs to improve the bioavailability of this polyphenol and enhance its uptake efficiency and cytotoxicity to breast cancer cells. The researchers deposited alginate and chitosan in a layer-by-layer manner on iron oxide (Fe_3_O_4_) magnetic NPs, obtaining final nanoconstructs in the 120–200 nm range. The as-synthesized nanomaterials have demonstrated targeted and sustained delivery of curcumin with the aid of a magnetic field, leading to up to 6-fold higher uptake efficiency than cancer cells treated with free drugs.

Another example is offered by Chen et al. [115], who have developed a self-healing hydrogel encapsulated with magnetic gelatin microspheres (MGMs). The scientists crosslinked carboxyethyl chitosan and oxidized alginate by the Schiff-base reaction, creating a scaffold for MGMs loaded with 5-fluoracil. The as-described delivery platform showed suitable mechanical and biological properties, excellent self-healing ability under physiological conditions, and sustained drug release, being considered promising for drug delivery purposes and soft tissue engineering.

### 4.2. Antimicrobial Agents

Bacterial infections are a common cause of hospitalization, while nosocomial infections are frequent issues within acute/critical care facilities worldwide [116,117,118]. The majority of these infections could be managed by administering antibiotic drugs, as many antimicrobial agents have been developed over the years. Nonetheless, it was observed that antibiotic misuse and/or overuse resulted in the emergence of antimicrobial resistance, leading to poor compliance to conventional treatments and more virulent infections [119,120,121,122].

In this context, the intrinsic antimicrobial properties of alginate and chitosan have gathered renewed interest for developing innovative antibiotic-free antimicrobial agents. This strategy is considered highly advantageous as it has the potential to fight against drug-resistant pathogens while avoiding antibiotic-associated side effects [123,124,125].

For instance, Yoncheva et al. [74] have incorporated oregano oil into chitosan-alginate NPs and tested them against eight microbial strains. These small-sized (320 nm) and negatively charged (−25 mV) particles have shown promising results against Gram-positive and Gram-negative bacteria, while in vitro cytotoxicity tests on human keratinocytes and in vivo skin irritation tests proved the safety profile of these nanosystems. Hence, the authors concluded that CANPs loaded with oregano oil could serve as a natural topical delivery system for treating microbial infections of the skin and other soft tissues.

Other natural-based cargos were used by Paiva et al. [126]. The authors loaded anacardic acid and cardol into chitosan-alginate core-shell NPs. The particles presented a slow-release rate that contributed to enhancing their inhibitory capacity against dermatophytes; the obtained results are considered promising for developing innovative antimicrobial control applications.

One more example is proposed by Bilal et al. [127], who have synthesized silver nanoparticles loaded in chitosan-alginate nanostructures. Their constructs exhibited a significant reduction in the log values of tested bacterial strains (i.e., *Staphylococcus aureus*, *Pseudomonas aeruginosa*, *Klebsiella pneumoniae*, *Acinetobacter baumannii*, *Morganella morganii*, and *Haemophilus influenza*). Moreover, cytotoxic activity has been noticed against HeLa cancer cells, suggesting the two-fold biomedical potentiality of the newly designed nanoparticles.

Similarly, Gomez Chabala et al. [128] have tackled the synergistic properties of silver NPs, chitosan, alginate, and *Aloe vera*. The developed nanosystem demonstrated antibacterial activity against *S. aureus* and *P. aeruginosa*, being a potential alternative for antibiotics to be used in wound dressings.

Nanomaterials with enhanced antimicrobial properties have also been obtained by encapsulating different antibiotics into chitosan-alginate-based nanoparticles. This approach was seen to improve the antibacterial activity of the drug while diminishing its associated cytotoxicity to human cells.

For example, Kumar et al. [129] have successfully loaded rifaximin into chitosan-alginate nanoparticles, obtaining nanoparticulate antimicrobial agents with outstanding antibacterial activities against *E. coli*, *P. aeruginosa*, and *Bacillus haynesii*. Similarly, Kadhum and Zaidan [130] have prepared CANPs loaded with doxycycline that exhibited superior effectiveness to the free drug, being suitable for the treatment of *Enterobacteriacae* infections at low concentrations of antibiotic.

Furthermore, Al-Getahami and Al-Qasmi [131] have obtained important results when loading camptothecin into calcium alginate-chitosan nanocomposites. The authors reported enhanced inhibitory activity against Gram-negative bacteria, which could be explained by the induction of genetic effects. The designed nanoplatform could cause point mutations in treated bacteria, disrupting DNA and protein synthesis biochemical pathways.

**Figure 7:**
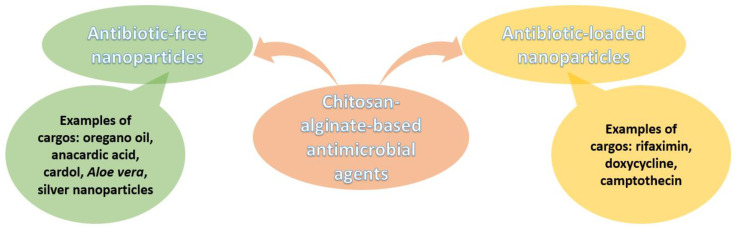
Overview of chitosan-alginate-based antimicrobial nanoformulations.

For clarity, Figure 7 summarizes the discussion on chitosan-alginate antimicrobial formulations in a clear visual manner.

### 4.3. Vaccine Adjuvants

Vaccination is a fundamental method of preventing virus pathogenicity, as it diminishes the burden associated with many infectious diseases. However, most vaccines face limitations in terms of degradation susceptibility, short duration of action, potential side effects, and inflammatory reactions at the administration site [47,132]. To avoid these drawbacks, vaccine formulations can be encapsulated into various nanocarriers, including chitosan-alginate-based vehicles. Moreover, the use of polymer-based nanovaccines brings a series of advantages, including strong cellular immune responses, sustained antigen release, anti-inflammatory responses, the possibility of needle-free dosage, and administration via various routes [133].

Onuigbo et al. [134] have coated oral fowl typhoid vaccine with chitosan-alginate microparticles, orally administered this formulation to birds, and compared the immune responses with the conventional subcutaneous administration of free-vaccine. Similar results were obtained for both formulations, with 100% protection and no mortality within the tested birds. Nonetheless, the polymeric coating showed protective efficacy of the oral route, preventing vaccine destruction in the gastrointestinal tract.

Giacomello et al. [135] have prepared chitosan-coated alginate microparticles for the oral delivery of immune-prophylactics to finfish. Investigations revealed that the in vivo administration by medicated food protects the microparticles while transiting the stomach, ensuring cargo release in the anterior intestines (i.e., the *villum sectum*—2 h after feeding, and the basal *lamina* of epithelial cells—4 h after feeding). The authors concluded that the as-designed immune-prophylaxis vaccines could replace antibiotics use in aquaculture.

Another oral vaccine delivery platform was designed by Biswas et al. [136]. The researchers formulated low molecular weight chitosan nanoparticles entrapped with measles antigen and coated with sodium alginate. Results showed that the engineered formulation induced a strong immune response in tested mice, protected the antigen in the gastric environment, and sustained release kinetics while maintaining low cytotoxicity. The coating strategy was also exploited by Yu et al. [137], who have used alginate-chitosan-coated LDH (layered double hydroxide NPs) nanocomposites as carriers for protein vaccine delivery.

Alternatively, alginate can also be combined with chitosan or trimethyl chitosan (TMC) to modify their immunostimulatory properties and increase their stability. For instance, Mosafer et al. [138] loaded inactivated PR8 influenza virus into chitosan or TMC NPs and coated them with sodium alginate. The authors reported that the PR8-TMC-alginate resulted in a significantly higher IgG2a/IgG1 ratio than the PR8-chitosan-alginate and PR8 free virus, which is an efficient intranasal antigen delivery system.

**Figure 8:**
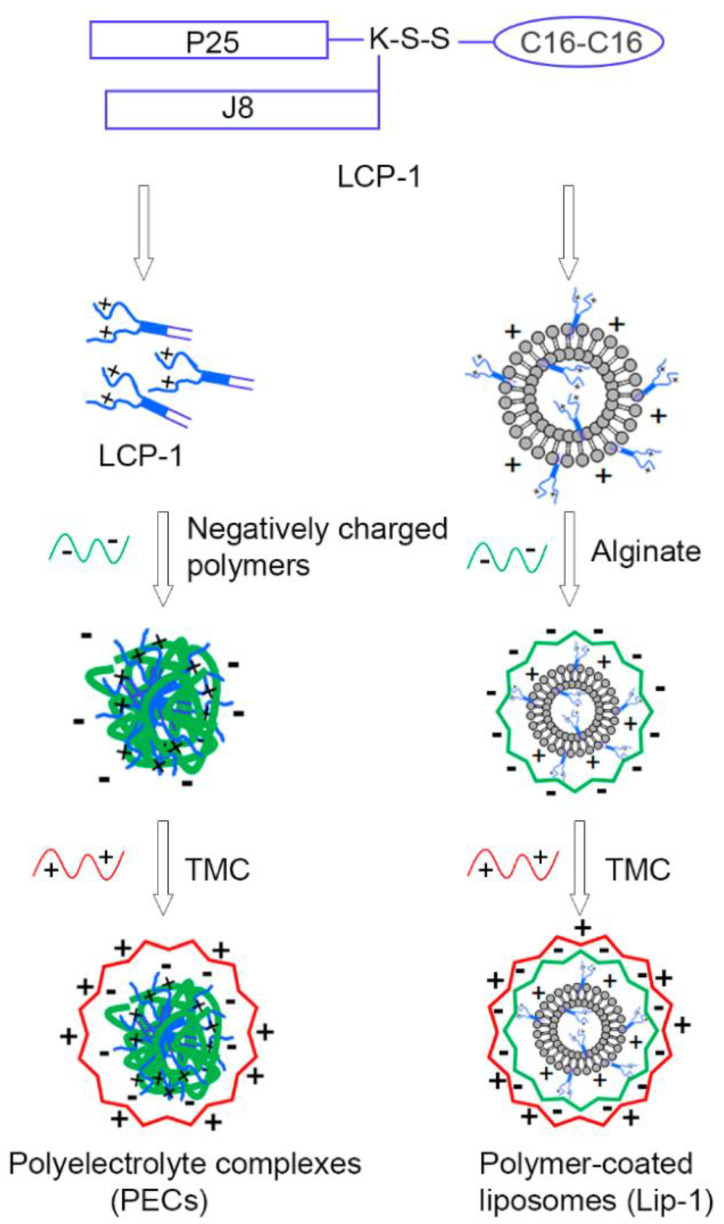
Schematic representation of the vaccine delivery systems developed by Zhao et al. [139]. Reprinted from an open-access source.

One more interesting study is proposed by Zhao et al. [139]. The authors developed two nanoplatforms for the nasal delivery of lipopeptide subunit vaccine (LCP-1) against group A streptococcus, namely LCP-1-loaded and alginate/TMC-coated liposomes (Lip-1) and LCP-1/alginate/TMC polyelectrolyte complexes (PEC-1) (Figure 8). It was observed that PEC-1 induced stronger humoral immune responses than Lip-1. Moreover, the liposome-free NPs were easier to prepare and were more cost-effective.

## 5. Other Applications

In addition to their promising biomedical applications, chitosan-alginate-based nanomaterials can be involved in various other uses. Their loading capacity, encapsulation efficiency, complexation ability, environmental-friendly manufacturing, and safe human use make them ideal candidates for applications in agriculture, the food industry, and water treatment. In this respect, several such applications are discussed in the following subsections.

### 5.1. Water Treatment

Industrial settings often lead to water pollution by bioaccumulating toxic metals that do not undergo natural biodegradation. Several methods, including ion exchange, chemical precipitation, electrochemical treatment, filtration, and adsorption, have been employed to remove such metals from the aqueous systems. Out of the enumerated possibilities, adsorption to a solid is one of the most economical strategies. Thus, the biodegradability and abundancy of alginate and chitosan have attracted attention for their use in heavy metal ions removal from water effluents [140].

In this regard, Dubey et al. [141] have prepared chitosan-alginate NPs for the effective and economically viable removal of Hg^2+^ ions. The maximum adsorption capacity of CANPs was noted in the following conditions: 5 pH, 200 mg adsorbent dose, 90 min contact time, 4 g/L initial metal ion concentration, and 30 °C. Moreover, the particles are able to desorb the ions in acidic pH, allowing their regeneration and reuse for further metal removal.

Similarly, Almutairi et al. [142] have studied the removal of hexavalent chromium (Cr^6+^) from polluted water by the use of negatively charged (−23.2 mV) chitosan-alginate nanocomposites incorporated with iron NPs. High efficiency was reported, the optimum conditions for Cr^6+^ adsorption being 5.0 pH, 4 g/L adsorbent dose, 210 min contact time, and 75 ppm initial Cr^6+^ concentration.

**Figure 9:**
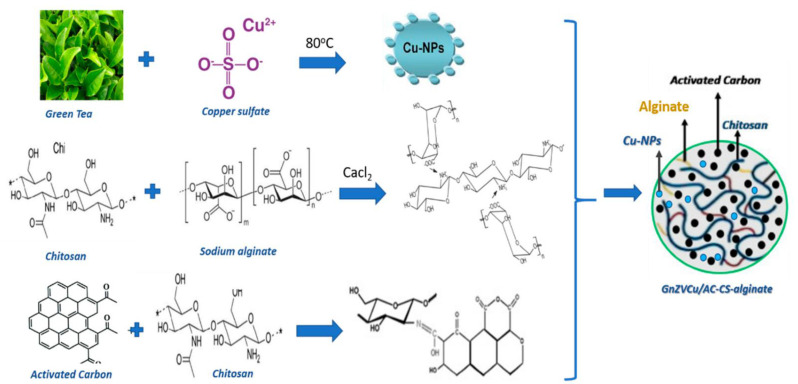
Schematic representation of synthesis mechanism and final structure of chitosan-alginate-based nanocomposite developed by Ahmed et al. [143]. Reprinted from an open-access source.

In contrast, Ahmed et al. [143] have developed more complex nanocomposites comprising green nano-zerovalent copper, activated carbon, chitosan, and alginate (Figure 9). These nanosystems were able to remove Cr^6+^ from polluted water in a proportion of up to 97.5%, with optimum adsorption at pH 2, being promising materials for water treatment.

### 5.2. Agricultural Applications

**Figure 10:**
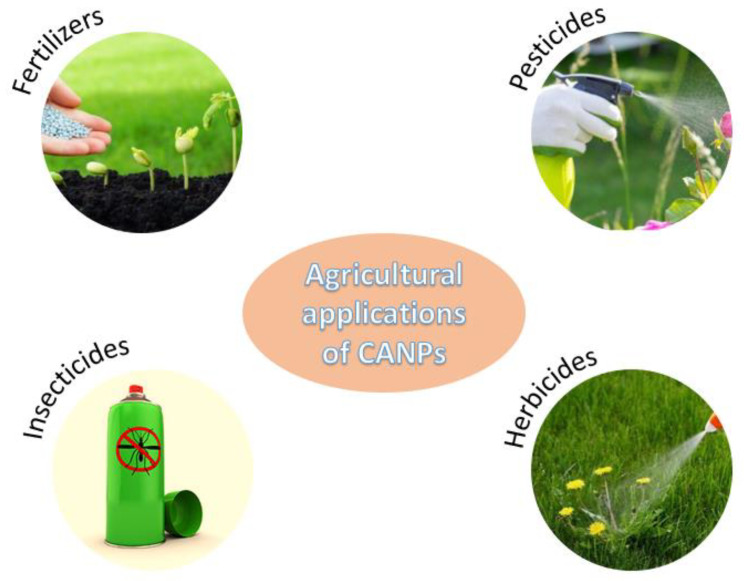
Examples of chitosan-alginate-based nanomaterials applications in agriculture.

Chitosan-alginate NPs have also found uses in agriculture, as depicted in Figure 10.

To meet the increasing global food demand, scientists have developed novel nanoscale fertilizers. In this respect, Leonardi et al. [144] have designed a shell made by polyelectrolyte complexation of chitosan and sodium alginate into which they incorporated copper oxide NPs. The nanosystem’s efficacy was evaluated on *Fortunella margarita* Swingle seeds, demonstrating benefits for seedling growth and development of epigean and hypogean parts. Thus, the authors concluded that the tested nanoparticles could serve as a smart delivery nanofertilizer produced by an eco-sustainable method.

Another interesting use of CANPs in agriculture is represented by the encapsulation of insecticides to diminish their adverse effect on the environment. In particular, Kaur et al. [145] have employed ionic gelation and polyelectrolyte complexation techniques to load cartap hydrochloride into chitosan-alginate nanospheres of ~100–170 nm size range. With an encapsulation efficiency higher than 75% and stability maintained for 30 days at ambient temperature, the developed nanosystem is considered promising for reducing field application frequency. Moreover, its slow release to the target organism is also economically relevant and safe for the environment.

Alternatively, Kumar et al. [146] have fabricated alginate-chitosan-based nanocapsules loaded with acetamiprid. The NPs were spherical, stable, with an encapsulation efficiency of 62% and a maximum pesticide release at alkaline pH. The as-described nanoformulation has the potential to reduce pesticides’ frequency of application by controlling the agrochemical release, subsequently reducing the associated side effects.

Herbicides can also be loaded into CANPs. Maruyama et al. [147] have encapsulated imazapic and imazapyr into chitosan-alginate NPs, improving their mode of action, reducing their toxicity to non-targeted organisms, and diminishing the risk of wider contamination. Similarly, Silva et al. [148] have loaded CANPs with paraquat and obtained reduced negative impacts compared to the free herbicide, as the nanocarrier altered the release profile and interaction with the soil.

### 5.3. Food Additives

The loading capacity of chitosan-alginate nanoparticles can also be exploited for designing novel food additives. For instance, Liu et al. [149] have incorporated ε-polylysine into spherical CANPs within the 200–500 nm size range. The researchers noticed a three times higher bacteriostatic activity of these nanoparticles than free ε-polylysine, concluding that the enhanced antibacterial activity recommends the newly developed nanosystem as a food preservative.

Another example is offered by Yoncheva et al. [74], who have designed chitosan-alginate nanoparticles encapsulated with oregano oil. The scientists reported a considerably enhanced antimetabolic activity than for the pure oil in several microbial strains, recommending this nanoformulation as a food additive with antimicrobial activity. Moreover, the authors suggest that their newly developed system should be tested for its antibacterial effect in vacuum-packed foods, accounting for their compatibility with the oregano oil taste and other organoleptic characteristics.

One more example for food microbial growth control is proposed by Zimet et al. [150]. The researchers fabricated nisin-loaded CANPs and reported that this nanoformulation was able to sustain its antimicrobial activity against *L. monocytogenes* for 21 days in vitro at 4 °C, and for up to 24 days in vacuum-sealed, refrigerated beef samples (as compared to 17 days for free nisin). Thus, the authors recommend the use of the designed NPs for their antimicrobial activity and ability to extend the shelf-life of lean beef.

A different application in the food industry has been reported by Ding et al. [151], who have created an innovative fish oil delivery system. The scientists prepared several multilayer emulsions of gelatin particles and chitosan-alginate shells through layer-by-layer electrostatic deposition, concluding that emulsion creaming stability during storage and the emulsion droplet stability against the gastric phase depend on the interfacial layer number. These alginate-chitosan-based delivery systems are considered promising for food products, such as food beverages and cheese, overcoming the fishy taste and poor water solubility of fish oils.

Another interesting use is proposed by Benucci et al. [152]. The researchers employed double-layer calcium alginate-chitosan microcapsules for entrapping yeast cells with application in sparkling wine production. By use of as-such immobilized yeasts, there can be accelerated and simplified riddling and subsequent disgorging procedures, while the sensory properties (i.e., aroma, taste, and body) remain similar to those produced by free yeasts.

## 6. Conclusions

In summary, chitosan-alginate-based nanoparticles’ unique structures and properties make them suitable for carrying numerous and varied cargos. Considering the recent research progress highlighted in this paper, CANPs hold great promise for creating advanced formulations for the biomedical and pharmaceutical domains. Furthermore, as CANPs-based systems have been developed for various administration routes, including oral, ocular, nasal, transdermal, and intravenous delivery, these nanocarriers can be considered key factors for efficiently treating many infectious and chronic diseases without imparting toxicity to healthy tissues. Nonetheless, additional research is required before translating these emerging nanosystems to the clinic, especially because, to date, most of the studies have been performed in vitro or/and on animal models.

Moreover, interesting possibilities can be envisaged in agriculture, the food industry, or water pollution management. Given that the tested chitosan-alginate nanoparticulate systems showed promising results, it can soon be expected that some of these formulations may enter the market as more environmentally friendly solutions than currently used fertilizers, pesticides, and herbicides or as more performant food additives.

In conclusion, current results should serve as an inception point for further research of novel and improved chitosan-alginate-based nanosystems, paving the way for implementation into the practice of smart nano-enabled products.

## Figures and Tables

**Table 1:** Examples of oral drug delivery systems based on chitosan-alginate nanoparticles.

Delivery System	Carried Drug	Synthesis Method	Physicochemical Properties	Applications	Ref.
CANPs	Curcumin diethyl diglutarate	Oil in water emulsification & ionotropic gelation	Size: 215 nm Zeta potential: −24.1 mV Encapsulation efficiency: 85% Loading capacity: 27% Composition: chitosan/alginate in a mass ratio of 0.065:1, Pluronic^®^F127 Stability: up to 3 months at 4 °C	Cancer treatment	[79]
CANPs	Curcumin diglutaric acid	Oil in water emulsification & ionotropic gelation	Size: 212–552 nm Zeta potential: from −17.2 to −29.2 mV Composition: chitosan/alginate in various mass ratio, Pluronic^®^F127	Cancer treatment	[80]
Succinylated chitosan-alginate core-shell-corona NPs	Quercetin	Ionic cross-linking	Minimum size: ~91.58 nm Encapsulation efficiency: ~95% Composition: succinyl chitosan, alginate	Diabetes treatment	[84]
Chitosan-coated alginate NPs	Insulin	Polyelectrolyte complexation & ionotropic gelation	Size: 340.40 ± 2.39 nm Encapsulation efficiency: 72.78 ± 1.25% Composition: alginate, Pluronic F-68, calcium chloride in various ratios + chitosan coating	Diabetes treatment	[82]
Polyelectrolyte complexes of chitosan-coated NPs and alginate-coated NPs	Insulin	Optimized double emulsion method	Composition: (*v*/*v* = 1:1) chitosan and alginate coated monomethoxy polyethylene glycolpoly (lactic-co-glycolic acid) (mPEG-b-PLGA) NPs Chitosan-coated NPs Size: 260.1 ± 17.1 nm Polydispersity index: 0.18 ± 0.10 Zeta potential: -55.7 ± 6.6 mV Encapsulation efficiency: 81.5 ± 7.4% Loading capacity: 10.7 ± 1.3% Alginate-coated NPs Size: 224.4 ± 13.18 nm Polydispersity index: 0.08 ± 0.05 Zeta potential: +13.7 ± 1.6 mV Encapsulation efficiency: 55.2 ± 7.0% Loading capacity: 4.9 ± 0.7%	Diabetes treatment	[83]
Alginate coated chitosan core-shell NPs	Naringenin	Sonication	Size: 216.44 ± 6 nm Zeta potential: −36.01 ± 2.7 mV Polydispersity index: 0.39 ± 0.14 Encapsulation efficiency: 91% Composition: alginate and chitosan at different weight ratios (optimum results for 1:3)	Diabetes treatment	[69]
CANPs	Lovastatin	Ionic gelation	Shape: spherical Size: 50–100 nm Composition: chitosan/alginate in a mass ratio of 3:6.5	Treatment of obesity and cardiovascular disease	[86]
CANPs	Rosuvastatin calcium	Ionotropic pre-gelation & polyelectrolyte complexation	Size: ~349.3 nm Zeta potential: ~+29.1 mV Composition: alginate, chitosan	Antilipidemic formulation	[85]
CANPs	Quercetin	Electrostatic gelation	Two types of nanoparticles: NP1 Size: ~350 nm Zeta potential: ~−30 mV Composition: chitosan/alginate with a higher concentration of alginate NP2 Size: ~550 nm Zeta potential: ~30 mV Composition: chitosan/alginate with a higher concentration of chitosan	Protection against oxidative stress	[72]
CANPs	Quercetin	Electrostatic gelation	Two types of nanoparticles: NP1 Size: ~350 nm Zeta potential: ~−40 mV Composition: chitosan/alginate with a higher concentration of alginate NP2 Size:~600 nm Zeta potential: ~35 mV Composition: chitosan/alginate with a higher concentration of chitosan	Therapeutic approach against oxidative stress-induced liver injury	[73]
Chitosan-alginate core-shell corona shaped NPs	Dimethyl fumarate	Ionotropic pre-gelation & polyelectrolyte complexation	Size: 561 ± 53.05 nm Zeta potential: −27.2 ± 6.33 mV Composition: chitosan, alginate	Multiple sclerosis treatment	[87]
Chitosan oligosaccharide/alginate NPs	Astaxanthin	Oil in water emulsification & ionotropic gelation	Size: 264 ± 32 nm Zeta potential: −22.1 ± 1.3 mV Encapsulation efficiency: 71.3 ± 2.2% Loading capacity: 6.9 ± 1.6% Composition: chitosan, alginate	Nutraceutical or functional foods	[88]

**Table 2:** Examples of ocular drug delivery systems based on chitosan-alginate nanoparticles.

Delivery System	Carried Drug	Synthesis Method	Physicochemical Properties	Applications	Ref.
Chitosan-coated alginate NPs	Daptomycin	Ionotropic pre-gelation & polyelectrolyte complexation	Size: 380–420 nm Encapsulation efficiency: 79–92% Composition: alginate, chitosan	Treatment of bacterial endophthalmitis	[96]
Chitosan-coated CANPs	5-Fluorouracil	Ionic gelation	Size: 329–505 nm Zeta potential: 18.5–28.9 mv Loading capacity: 2.68–18.93% Encapsulation efficiency: 6.19–26.66% Composition: alginate, chitosan	Conjunctival/ corneal squamous cell carcinoma	[97]
Chitosan-oleic acid-sodium alginate NPs	Lutein	Ionic gelation	Size: 40–160 nm Polydispersity index: 0.174 ± 0.02 Zeta potential: 45 ± 5 mV Composition: chitosan, oleic acid, alginate	Therapeutic approach against macular degeneration and diabetic retinopathy	[98]
Chitosan-alginate microspheres	Azelastine hydrochloride	Modified ionic gelation	Size: 3.55–6.70 μm Zeta potential: 24.55-49.56 mV Maximum loading capacity: 73.05% Composition: chitosan, alginate	Conjunctivitis treatment	[99]
CANPs	Ofloxacin	Modified ionotropic gelation	Size: 113.8–509 nm Zeta potential: 16.2–40.3 mV Encapsulation efficiency: 19.7–33.1% Composition: chitosan, alginate, tripolyphopsphatesodium	Eye infections	[100]
Chitosan-coated alginate NPs	Betamethasone sodium phosphate	Electrospraying & emulsification	Size: 150–300 nm Loading capacity: 7% Encapsulation efficiency: 40% Composition: alginate, chitosan	Eye diseases	[71]
Thiolated chitosan and sodium alginate NPs	Fluorescein isothiocyanate	Modified ionic gelation	Size: 408.0 ± 6.4 nm Polydispersity index: 0.34 ± 0.07 Zeta potential: 49.2 ± 2.3 mV Encapsulation efficiency: 92.1 ± 1.9% Composition: thiolated chitosan, alginate	Eye diseases	[101]

**Table 3:** Examples of chitosan-alginate nanoparticles for various administration routes.

Delivery System	Carried Drug	Synthesis Method	Physicochemical Properties	Administration Route	Applications	Ref.
CANPs	Bee venom	Oil in water emulsification	Size: 434.6 ± 22.1 nm Polydispersity index: 0.433 ± 0.016 Loading capacity: 3.3 ± 0.08% Encapsulation efficiency: 46.7 ± 2.14% Composition: chitosan, alginate	Nasal	PRRSV	[102]
CANPs	Rifampicin Ascorbic acid	Ionic gelation	Size: 300 ± 30 nm Polydispersity index: 0.22 ± 0.03 Zeta potential: −31.4 ± 0.8 mV Loading capacity: 24 ± 1% Encapsulation efficiency: 50 ± 1% Composition: low viscosity sodium alginate, low molecular weight chitosan	Intratracheal	*S. aureus* infections	[103]
CANPs	Doxorubicin	Electrostatic gelation	Shape: spherical Size: ~300 nm Zeta potential: from −22.5 to −25.0 mV Encapsulation efficiency: >90% Composition: chitosan/alginate in a mass ratio of 1:10	Intravenous	Melanoma treatment	[105]
Chitosan-alginate-sodium tripolyphosphate (STPP) NPs	Curcumin	Sonication	Shape: spherical Size: ~50 nm Composition: chitosan, alginate, STPP	Intravenous	Epilepsy treatment	[70]
CANPs	Pirfenidone	Pre-gelation	Shape: spherical Size: ~80 nm Loading capacity: 50% Encapsulation efficiency: 94% Composition: chitosan, alginate	Transdermal	Treatment of idiopathic pulmonary fibrosis	[104]
Mal-CS-Cat-Alg NPs	Doxorubicin	Ionic gelation & sonication	Shape: spherical Size: 116–182 nm Maximum loading capacity: 74.7 ± 0.3% Composition: 6-maleimidohexanoic acid, chitosan, N-Ethyl-N′−(3-dimethylaminopropyl) carbodiimide hydrochloride, N-hydroxysuccinimide	Intravesical	Bladder cancer treatment	[106]
Chitosan-coated alginate microcapsules	Probiotics	Extrusion of polymer dispersions & polyelectrolyte complexations	Shape: spherical Size: 125–150 μm Zeta potential: 1–12 mV Composition: alginate, chitosan	Periodontal	Periodontal disease	[75]

**Table 4:** Examples of in vitro-tested drug delivery systems based on chitosan-alginate nanoparticles.

Delivery System	Carried Drug	Synthesis Method	Physicochemical Properties	Applications	Ref.
CANPs	Endolysin	Ionotropic pre-gelation & polyelectrolyte complexation	Hydrodynamic diameter: 276.5 ± 42 nm Polydispersity index: 0.34 ± 0.02 Zeta potential: −25 mV Composition: alginate, chitosan	Staphylococcal infections	[107]
CANPs stabilized with carrageenan	Ethionamide	Ionotropic gelation	Shape: spherical Size: ~300 nm Composition: chitosan, alginate, carrageenan in different percentages (i.e., 0%, 42%, and 59%)	Tuberculosis treatment	[10]
CANPs	Curcumin	Oil in water emulsification & ionotropic gelation	Size: ~400 nm Zeta potential: ~−22 mV Composition: chitosan/alginate in a mass ratio of 0.08:1	Photodynamic therapy system for psoriasis treatment	[108]
CANPS	Crocin	Ultrasonic-mediated synthesis	Size: 236 nm Polydispersity index: 0.476 Encapsulation efficiency: 38.16% Composition: 0.08 *w*/*v* chitosan, 0.10% *w*/*v* alginate	Cancer treatment	[109]
Chitosan-alginate-STPP NPs	Curcumin	Ultrasonic-assisted synthesis	Shape: spherical Size: ~50 nm Encapsulation efficiency: ~70% Composition: chitosan, alginate, STPP	Cancer treatment	[110]
CANPs	Doxorubicin	Ionotropic gelation	Size: ~300 nm Polydispersity index: 0.2 Loading capacity: ~18% Encapsulation efficiency: ~99% Composition: chitosan, alginate	Lymphoma treatment	[111]
Alginate-coated chitosan hollow nanospheres	Doxorubicin Paclitaxel	Hard template method	Diameter: ~150 nm Wall thickness: 20 nm Paclitaxel:doxorubicin loading ratio: 1:7 Composition: chitosan, alginate	Lung cancer treatment	[112]
CANPs	Quercetin	Ionotropic gelation & ultrasonication	Shape: rod-like Size: ~118–255 nm Loading capacity: 46.5% Encapsulation efficiency: 82.4% Composition: chitosan, alginate	Pharmaceutical applications	[113]

## Data Availability

The data presented in this study are available on request from the corresponding author.
